# Learning a peptide-protein binding affinity predictor with kernel ridge regression

**DOI:** 10.1186/1471-2105-14-82

**Published:** 2013-03-05

**Authors:** Sébastien Giguère, Mario Marchand, François Laviolette, Alexandre Drouin, Jacques Corbeil

**Affiliations:** 1 Department of Computer Science and Software Engineering, Université Laval, Québec, Canada; 2 Department of Molecular Medicine, Université Laval, Québec, Canada

## Abstract

**Background:**

The cellular function of a vast majority of proteins is performed through physical interactions with other biomolecules, which, most of the time, are other proteins. Peptides represent templates of choice for mimicking a secondary structure in order to modulate protein-protein interaction. They are thus an interesting class of therapeutics since they also display strong activity, high selectivity, low toxicity and few drug-drug interactions. Furthermore, predicting peptides that would bind to a specific MHC alleles would be of tremendous benefit to improve vaccine based therapy and possibly generate antibodies with greater affinity. Modern computational methods have the potential to accelerate and lower the cost of drug and vaccine discovery by selecting potential compounds for testing in silico prior to biological validation.

**Results:**

We propose a specialized string kernel for small bio-molecules, peptides and pseudo-sequences of binding interfaces. The kernel incorporates physico-chemical properties of amino acids and elegantly generalizes eight kernels, comprised of the Oligo, the Weighted Degree, the Blended Spectrum, and the Radial Basis Function. We provide a low complexity dynamic programming algorithm for the exact computation of the kernel and a linear time algorithm for it’s approximation. Combined with kernel ridge regression and SupCK, a novel binding pocket kernel, the proposed kernel yields biologically relevant and good prediction accuracy on the PepX database. For the first time, a machine learning predictor is capable of predicting the binding affinity of any peptide to any protein with reasonable accuracy. The method was also applied to both single-target and pan-specific Major Histocompatibility Complex class II benchmark datasets and three Quantitative Structure Affinity Model benchmark datasets.

**Conclusion:**

On all benchmarks, our method significantly (p-value ≤ 0.057) outperforms the current state-of-the-art methods at predicting peptide-protein binding affinities. The proposed approach is flexible and can be applied to predict any quantitative biological activity. Moreover, generating reliable peptide-protein binding affinities will also improve system biology modelling of interaction pathways. Lastly, the method should be of value to a large segment of the research community with the potential to accelerate the discovery of peptide-based drugs and facilitate vaccine development. The proposed kernel is freely available at http://graal.ift.ulaval.ca/downloads/gs-kernel/.

## Background

The cellular function of a vast majority of proteins is performed through physical interactions with other proteins. Indeed, essentially all of the known cellular and biological processes depend, at some level, on protein-protein interactions (PPI) [1,2]. Therefore, the controlled interference of PPI with chemical compounds provides tremendous potential for the discovery of novel molecular tools to improve our understanding of biochemical pathways as well as the development of new therapeutic agents [3,4].

Considering the nature of the interaction surface, protein secondary structures are essential for binding specifically to protein interaction domains. Peptides also represent templates of choice for mimicking a secondary structure in order to modulate protein-protein interactions [5,6]. Furthermore, they are a very interesting class of therapeutics since they display strong activity, high selectivity, low toxicity and fewer drug-drug interactions. They can also serve as investigative tools to gain insight into the role of a protein, by binding to distinct regulatory regions to inhibit specific functions.

Yearly, large sums of money are invested in the process of finding druggable targets and identifying compounds with medicinal utility. The widespread use of combinatorial chemistry and high-throughput screening in the pharmaceutical and biotechnology industries implies that millions of compounds can be tested for biological activity. However, screening large chemical libraries generates significant rates of both false positives and negatives. The process is expensive and faces a number of challenges in testing candidate drugs and validating the hits, all of which must be done efficiently to reduce costs and time. Computational methods with reasonable predictive power can now be envisaged to accelerate the process, thus providing an increase in productivity at a reduced cost.

As an example, peptides ranging from 8 to 12 AA represent the recognition unit for the MHC (Major Hiscompatibility Complex). Being capable of predicting which peptides bind to a specific MHC alleles would be of tremendous benefit to improve vaccine based therapy, possibly generating antibodies with greater affinity that could yield an improved immune response. Moreover, simply having data on the binding affinity of peptides and proteins could readily assist system biology modelling of interaction pathways.

The ultimate goal is to build a predictor of the highest binding affinity peptides. This task would be facilitated if one had a fast and accurate binding affinity predictor. Indeed, with this predictor, one could easily predict the binding affinity of huge sets of peptides and select the candidates with the highest predicted binding affinity, or use stochastic search methods such as simulated annealing if the set of peptides were too large. This paper provides a step in this direction with the use of a machine learning algorithm based on kernel methods and a novel kernel.

Traditional machine learning approaches focused on using binary binding data for classification of compounds (binding, non-binding) [7,8]. Non-binding compounds are rarely known and valuable quantitative binding affinity information is lost during training, a major obstacle to binary classification. Other approaches used information from the US Food and Drug Administration’s adverse event reporting system for the prediction of off-target protein interactions [9]. These methods can predict unknown drug-target interactions from FDA approved drugs but are not suited for the identification of new pharmaceutical compounds. New databases, such as the PepX database, contain binding affinities between peptides and a large group of protein families. The first part of this paper presents a general method for learning a binding affinity predictor between any peptide and any protein, a novel machine learning contribution to biology.

The Immune Epitope Database (IEDB) [10] contains a large number of binding affinities between peptides and Major Histocompatibility Complex (MHC) alleles. Predicting methods for MHC class I alleles have already obtained great success [8,11]. The simpler binding interface of MHC-I molecules makes the learning problem significantly easier than for MHC-II molecules. Allele specific (single-target) methods for MHC class II alleles have also reasonable accuracy, despite requiring a large number of training examples for every allele in order to achieve adequate accuracy [11]. Pan-specific (multi-target) methods, such as MultiRTA [12] and NetMHCIIpan-2.0 [13], were designed in order to overcome this issue. These methods can predict, with reasonable accuracy, the binding affinity of a peptide to any MHC allele, even if this allele has no known peptide binders.

We propose a new machine learning approach based on kernel methods [14] capable of both single-target and multi-target (pan-specific) prediction. We searched for kernels that encode relevant binding information for both proteins and peptides. Therefore, we propose a new kernel, a Generic String (GS) kernel, that generalizes most of kernels currently used in this setting (RBF [14], Blended spectrum [14], Oligo [15], Weighted Degree [16],...). The GS kernel is shown to be a suitable similarity measure between peptides and pseudo-sequences of MHC-II binding interfaces.

For the machine learning algorithm itself, we show that kernel ridge regression [14] (KRR) is generally preferable to the support vector regression (SVR) algorithm [17] because KRR has less hyperparameters to tune than SVR, thus making the learning time smaller. The regression score obtained with the PepX examples is competitive with the ones generated on data sets containing peptides binding to a single protein, even if the former task is, in theory, much more difficult. For the peptide-MHC binding problem, comparison on benchmark datasets show that our algorithm surpasses NetMHCIIpan-2.0 [13], the current state-of-the-art method. Indeed, in the most difficult pan-specific case (when the algorithm is trained on all alleles except the allele used for testing), our algorithm performs better than the state of the art in most cases. Finally, we have found that ridge regression outperforms SVR on three quantitative structure affinity model (QSAM) single-target predictions benchmarks [18]. We thus propose a machine learning approach to immunology and a novel string kernel which have shown to yield impressive results on benchmark datasets for various biological problems.

## Methods

### Statistical machine learning and kernel ridge regression in our context

Given a set of training examples (or cases), the task of a learning algorithm is to build an accurate predictor. In this paper, each example will be of the form ((**x**, **y**), *e*), where **x** represents a peptide, **y** represents a protein, and *e* is a real number representing the binding energy (or the binding affinity) between the peptide **x** and the protein **y**. A multi-target predictor is a function *h* that returns an output *h*(**x**, **y**) when given any input (**x**, **y**). In our setting, the output *h*(**x**, **y**) is a real number estimate of the “true” binding energy (or the binding affinity) *e* between **x** and **y**. The predictor *h* is accurate on example ((**x**, **y**), *e*) if the predicted output *h*(**x**, **y**) is very similar to the real output *e*. A predictor is good when it is accurate on most future examples unseen during training.

With kernel methods, each input (**x**, **y**) is implicitly mapped to a *feature vector****ϕ***(**x**, **y**) = (*ϕ*_1_(**x**, **y**), *ϕ*_2_(**x**, **y**), …, *ϕ*_*d*_(**x**, **y**)) of large dimensionality *d*. Moreover, the predictor is represented by a real-valued weight vector **w** that lies in the space of feature vectors. Given an arbitrary input (**x**, **y**), the output of the predictor *h*_**w**_ is given by the scalar product

$$h_{w}(x,y)=w\cdot \phi (x,y)\overset{\text{def}}{=}\overset{d}{\underset{i=1}{\sum }}w_{i}\phi_{i}(x,y)\;.$$

The loss incurred by predicting a binding energy *h*_**w**_(**x**, **y**) on input (**x**, **y**), when the true binding energy is *e*, is measured by a *loss functionℓ*(**w**, (**x**, **y**), *e*). As is usual in regression, we will use the quadratic loss function

$$\ell (w,(x,y),e)={\left(e-w\cdot \phi (x,y)\right)}^{2}\;.$$

The fundamental assumption in machine learning is that each example ((**x**, **y**), *e*) is drawn according to some unknown distribution *D*. Then the task of the learning algorithm is to find the predictor *h*_**w**_ having the smallest possible *riskR*(*h*_**w**_) defined as the expected loss

$$R(h_{w})\overset{\text{def}}{=}\underset{((x,y),e)∼D}{E}\ell (w,(x,y),e)\;.$$

However, the learning algorithm does not have access to *D*. Instead, it has access to a training set $S\overset{\text{def}}{=}\{((x_{1},y_{1}),e_{1}),((x_{2},y_{2}),e_{2}),\ldots ,((x_{m},y_{m}),e_{m})\}$ of *m* examples where each example ((**x**_*i*_, **y**_*i*_), *e*_*i*_) is assumed to be generated independently according to the same (but unknown) distribution *D*. Modern statistical learning theory [14,19] tells us that the predictor *h*_**w**_ minimizing the *ridge regression cost functionF*(*S*, **w**) will have a small risk *R*(*h*_**w**_) whenever the obtained value of *F*(*S*, **w**) is small. Here, *F*(*S*, **w**) is defined as

$$\begin{array}{lll} F(S,w) & \overset{\text{def}}{=} & ∥w{∥}^{2}+C\overset{m}{\underset{i=1}{\sum }}\ell (w,(x_{i},y_{i}),e_{i})\\ & \;= & ∥w{∥}^{2}+C\overset{m}{\underset{i=1}{\sum }}{\left(e_{i}-w\cdot \phi (x_{i},y_{i})\right)}^{2}\;, \end{array}$$

for some suitably-chosen constant *C* > 0. The first term of *F*(*S*, **w**), $∥w{∥}^{2}\overset{\text{def}}{=}w\cdot w$, which is the squared Euclidean norm of **w**, is called a *regularizer* and it penalizes predictors having a large norm (complex predictors). The second term measures the accuracy of the predictor on the training data. Consequently, the parameter *C* controls the complexity-accuracy trade-off. Its value is usually determined by measuring the accuracy of the predictor on a separate (“hold-out”) part of the data that was not used for training, or by more elaborate sampling methods such as cross-validation.

The *representer theorem*[14,19] tells us that the predictor **w**^∗^ that minimizes *F*(*S*, **w**) lies in the linear subspace span by the training examples. In other words, we can write

$$w^{*}=\overset{m}{\underset{i=1}{\sum }}\alpha_{i}\phi (x_{i},y_{i})\;,$$

where the coefficients *α*_*i*_ are called the *dual* variables and provide collectively the dual representation of the predictor. This change of representation makes the cost function dependent on ***ϕ***(**x**_*i*_, **y**_*i*_) only via the scalar product $\phi (x_{i},y_{i})\cdot \phi (x_{j},y_{j})\overset{\text{def}}{=}k((x_{i},y_{i}),(x_{j},y_{j}))$ for each pair of examples. The function *k* is called a *kernel* and has the property of being efficiently computable for many feature maps ***ϕ***, even if the feature space induced by ***ϕ*** has an extremely large dimensionality. By using *k* instead of ***ϕ***, we can construct linear predictors in feature spaces of extremely large dimensionality with a running time that scales only with the size of the training data (with no dependence on the dimensionality of ***ϕ***). This fundamental property is also known as the *kernel trick*[14,19]. It is important to point out that, since a kernel corresponds to a scalar product in a feature space, it can be considered as a similarity measure. A large (positive) value of the kernel normally implies that the corresponding feature vectors point in similar directions, although a value close to zero normally implies that the two feature vectors are mostly orthogonal (dissimilar).

As was proposed by several authors [7,8,20,21], we restrict ourselves to joint feature maps having the form $\phi (x,y)=\phi_{X}(x)⊗\phi_{Y}(y)$ where ⊗ denotes the tensor product. The tensor product between two vectors **a** = (*a*_1_, …, *a*_*n*_) and **b** = (*b*_1_, …, *b*_*m*_) denotes the vector **a** ⊗ **b** = (*a*_1_*b*_1_, *a*_1_*b*_2_, …, *a*_*n*_*b*_*m*_) of all the *nm* products between the components of **a** and **b**. If we now define the peptide kernel $k_{X}$ by $k_{X}(x,x^{\prime })\overset{\text{def}}{=}\phi_{X}(x)\cdot \phi_{X}(x^{\prime })$, and the protein kernel $k_{Y}$ by $k_{Y}(y,y^{\prime })\overset{\text{def}}{=}\phi_{Y}(y)\cdot \phi_{Y}(y^{\prime })$, the joint kernel *k* simply decomposes as the product of $k_{X}$ and $k_{Y}$ because

() $$\begin{array}{llll} k((x,y),(x^{\prime },y^{\prime }))\; & \overset{\text{def}}{=} & \;\phi (x,y)\cdot \phi (x^{\prime },y^{\prime }) & \\ \; & \;= & \;\phi_{X}(x)⊗\phi_{Y}(y)\cdot \phi_{X}(x^{\prime })⊗\phi_{Y}(y^{\prime }) & \\ \; & \;= & \;(\phi_{X}(x)\cdot \phi_{X}(x^{\prime }))(\phi_{Y}(y)\cdot \phi_{Y}(y^{\prime })) & \\ \; & \overset{\text{def}}{=} & \;k_{X}(x,x^{\prime })k_{Y}(y,y^{\prime })\;. & \end{array}$$

Consequently, from the representer theorem we can write the multi-target predictor as

$$\begin{array}{lll} h_{w^{*}}(x,y) & =w^{*}\cdot \phi (x,y)=w^{*}\cdot (\phi_{X}(x)⊗\phi_{Y}(y))\; & \;\\ & =\overset{m}{\underset{i=1}{\sum }}\alpha_{i}k_{X}(x_{i},x)k_{Y}(y_{i},y)\;.\; & \; \end{array}$$

In the case of the quadratic loss *ℓ*(**w**, (**x**, **y**), *e*) = (*e* - **w** · *ϕ*(**x**, **y**))^2^, *F*(*S*, **w**) is a strongly convex function of **w** for any strictly positive *C*. In that case, there exists a single local minimum which coincides with the global minimum. This single minimum is given by the point **w**^∗^ where the gradient *∂F*(*S*, **w**) / *∂***w** vanishes. For the quadratic loss, this solution **w**^∗^ is given by

(1) $$\alpha ={\left(K+\frac{1}{C}I\right)}^{-1}e\;,$$

where $\alpha \;\overset{\text{def}}{=}\;(\alpha_{1},\ldots ,\alpha_{m})$, $e\;\overset{\text{def}}{=}\;(e_{1},\ldots ,e_{m})$, **K** denotes the Gram matrix of kernel values $K_{i,j}=k_{X}(x_{i},x_{j})k_{Y}(y_{i},y_{j})$, and **I** denotes de *m* × *m* identity matrix. Hence, the learning algorithm for kernel ridge regression just consists at solving Equation (1). Note that for a symmetric positive semi-definite kernel matrix **K**, the inverse of **K** + **I** / *C* always exists for any finite value of *C* > 0. Note also that the inverse of an *m* × *m* matrix is obtained in *O*(*m*^3^) time with the Gaussian-elimination method and in *O*(*m*^2.376^) time with the Coppersmith-Winograd algorithm.

Finally, we will also consider the single protein target case where only one protein *y* is considered. In this case, the predictor *h*_**w**_ predicts the binding energy from a feature vector $\phi_{X}$ constructed only from the peptide. Hence, the predicted binding energy for peptide **x** is now given by $w\cdot \phi_{X}(x)$. So, in this single protein target case, the cost function to minimize is still given by *F*(*S*,**w**) but with ***ϕ***(**x**, **y**) replaced by $\phi_{X}(x)$. Consequently, in this case, the solution is still given by Equation (1) but with a kernel matrix **K** given by $K_{i,j}=k_{X}(x_{i},x_{j})$. The single-target predictor is thus given by

$$h_{w^{*}}(x)=w^{*}\cdot \phi_{X}(x)=\overset{m}{\underset{i=1}{\sum }}\alpha_{i}k_{X}(x_{i},x)\;.$$

Kernel methods have been extremely successful within the last decade, but the choice of the kernel is critical for obtaining good predictors. Hence, confronted with a new application, we must be prepared to design an appropriate kernel. The next subsections show how we have designed and chosen both peptide and protein kernels.

### A generic string (GS) kernel for small bio-molecule strings

String kernels for bio-molecules have been applied with success in bioinformatics and computational biology. Kernels for large bio-molecules, such as the local-alignment kernel [22] have been well studied and applied with success to problems such as protein homology detection. However, we observed that these kernels perform rather poorly on smaller compounds (data not shown). Consequently, kernels designed for smaller bio-molecules like peptides and pseudo sequences have recently been proposed. Some of these kernels [15] exploit sub-string position uncertainty while others [23] use physicochemical properties of amino acids. We present a kernel for peptides that exploits both of these properties in a unified manner.

The proposed kernel, which we call the generic string (GS) kernel, is a similarity measure defined for any pair (**x**, **x**^′^) of strings of amino acids. Let *Σ* be the set of all amino acids. Then, given any string **x** of amino acids (e.g., a peptide), let |**x**| denote the length of string **x**, as measured by the number of amino acids in **x**. The positions of amino acids in **x** are numbered from 1 to |**x**|. In other words, **x** = *x*_1_, *x*_2_, …, *x*_|**x**|_ with all *x*_*i*_ ∈ *Σ*.

Now, let $\psi :\Sigma \to R^{d}$ be an encoding function such that for each amino acid *a*,

(2) $$\psi (a)=(\psi_{1}(a),\psi_{2}(a),\ldots \psi_{d}(a))$$

is a vector where each component *ψ*_*i*_(*a*) encodes one of the *d* properties (possibly physicochemical) of amino acid *a*. In a similar way, we define $\psi^{l}:\Sigma^{l}\to R^{\text{dl}}$ as an encoding function for strings of length *l*. Thus, ***ψ***^*l*^(**a**) encodes all *l* amino acids of **a** concatenning *l* vectors, each of *d* components:

(3) $$\psi^{l}(a_{1},a_{2},\mathrm{..},a_{l})\;\overset{\text{def}}{=}\;(\psi (a_{1}),\psi (a_{2}),\ldots ,\psi (a_{l}))$$

Let *L* ≥ 1 be a maximum length for substring comparison. We define the generic string (GS) kernel as the following similarity function over any pair (**x**, **x**^′^) of strings of length at least *L*:

(4) $$\begin{array}{l} \;\text{GS}(x,x^{\prime },L,\sigma_{p},\sigma_{c})\\ \overset{\text{def}}{=}\overset{L}{\underset{l=1}{\sum }}\overset{|x|-l}{\underset{i=0}{\sum }}\overset{|x^{\prime }|-l}{\underset{j=0}{\sum }}\;e^{\left(\frac{-{\left(i-j\right)}^{2}}{2\sigma_{p}^{2}}\right)}\;e^{\left(\frac{-∥\psi^{l}(x_{i+1},\mathrm{..},x_{i+l})\;-\;\psi^{l}(x_{j+1}^{\prime },\mathrm{..},x_{j+l}){∥}^{2}}{2\sigma_{c}^{2}}\right)}. \end{array}$$

In other words, this kernel compares each substring *x*_*i*+1_, *x*_*i*+2_ ,.., *x*_*i*+*l*_ of **x** of size *l* ≤ *L* with each substring $x_{j+1}^{\prime },x_{j+2}^{\prime }\mathrm{...},x_{j+l}^{\prime }$ of **x**^′^ having the same length. Each substring comparison yields a score that depends on the ***ψ***-similarity of their respective amino acids and a shifting contribution term that decays exponentially rapidly with the distance between the starting positions of the two substrings. The *σ*_*p*_ parameter controls the shifting contribution term. The *σ*_*c*_ parameter controls the amount of penalty incurred when the encoding vectors ***ψ***^*l*^(*x*_*i*+1_ ,.., *x*_*i*+*l*_) and $\psi^{l}(x_{j+1}^{\prime },\mathrm{..},x_{j+l}^{\prime })$ differ as measured by the squared Euclidean distance between these two vectors. The GS kernel outputs the sum of all the substring-comparison scores.

Also, note that the GS kernel can be used on strings of different lengths, which is a great advantage over a localized string kernel (of fixed length) such as the RBF, the weighted degree kernels [16,23] or KISS [8], a well known kernel method for the prediction of peptides binding to MHC-I. In fact, the GS kernel generalizes eight known kernels. Table 1 lists them with the fixed and free parameters. For example, when *σ*_*p*_ approaches + *∞* and *σ*_*c*_ approaches 0, the GS kernel becomes identical to the blended spectrum kernel [14], which has a free parameter *L* representing the maximum length of substrings. The free parameter values are usually determined by measuring the accuracy of the predictor on a separate (“hold-out”) part of the data that was not used for training, or by more elaborate sampling methods such as cross-validation.

**Table 1:** Special cases of the GS kernel

**Fixed parameters**	**Freeparameters**	**Kernel name**
*L* = 1, *σ*_*p*_ → 0, *σ*_*c*_ → 0		Hamming distance
*L* → *∞*, *σ*_*p*_ → 0, *σ*_*c*_ → 0		Dirac delta
*σ*_*p*_ → *∞*, *σ*_*c*_ → 0	*L*	Blended Spectrum [14]
*σ*_*p*_ → *∞*	*L*, *σ*_*c*_	Blended Spectrum RBF [23]
*σ*_*c*_ → 0	*L*, *σ*_*p*_	Oligo [15]
*L* → *∞*, *σ*_*p*_ → 0	*σ*_*c*_	Radial Basis Function (RBF)
*σ*_*p*_ → 0, *σ*_*c*_ → 0	*L*	Weighted degree (⋆) [16]
*σ*_*p*_ → 0	*L*, *σ*_*c*_	Weighted degree RBF (⋆) [23]
	*L*, *σ*_*p*_, *σ*_*c*_	Generic String (GS)

(⋆) Substituting ***ψ***^*l*^ by $\psi^{l}\sqrt{-\ln\beta_{l}}$ where the *β*_*l*_’s are the weighted degrees defined in [16]. Eight known kernels can be obtained by fixing different parameters of the GS kernel.

In contrast, Leslie et al. [24] proposed the mismatch kernel which also extends the spectrum kernel, adding the important notion of mismatches (mutations) in the comparison of k-mers. This was motivated by the fact that mutations occur in proteins and thus k-mers should be considered up to a certain amount of mismatches. Not all mutations are equal, some will not affect the function of a protein as others will dramatically change the conformation of a protein or the binding affinity of a peptide. This is the motivating idea behind the ***ψ*** encoding function, amino acids properties are used to have a smooth transition between unimportant and critical mutations. Moreover, the transition can be adjusted thought the *σ*_*c*_ parameter.

Also, Saigo et al. [22] proposed the local alignment (LA) kernel which sums all possible alignments with gaps between two sequences. The LA kernel is closely related to the popular Smith-Waterman alignment algorithm. In contrast, the GS kernel sums the contributions of all substrings according to their physicochemical properties with a position uncertainty penalising term. Also, the gap penalisation in the LA is well adapted to protein similarity by incorporating biological knowledge about protein evolution but not so much for identifying localized signals in sequences. Indeed, a small gap of only one amino acid in a peptide will have a dramatic influence on its contacting residues and therefore on its binding affinity. Finally, the LA kernel suffers from diagonal dominance, an issue the authors got around by taking the logarithm of the kernel. Unfortunately this operation does not preserve the positive definiteness of the kernel. However, the GS kernel does not suffer form diagonal dominance, thus avoiding many workarounds.

In the next subsection, we prove that the GS kernel is symmetric positive semi-definite and, therefore, defines a scalar product in some large-dimensional feature space (see [14]). In other words, for any hyperparameter values (*L*, *σ*_*p*_, *σ*_*c*_), there exists a function $\phi_{X_{\left(L,\sigma_{p},\sigma_{c}\right)}}$ transforming each finite sequence of amino acids into a vector such that

$$\text{GS}(x,x^{\prime },L,\sigma_{p},\sigma_{c})=\phi_{X_{\left(L,\sigma_{p},\sigma_{c}\right)}}(x)\cdot \phi_{X_{\left(L,\sigma_{p},\sigma_{c}\right)}}(x^{\prime })\;.$$

Consequently, the solution minimizing the ridge regression functional *F*(*S*, **w**) will be given by Equation (1) and is guaranteed to exist whenever the GS Kernel is used.

#### Symmetric positive semi-definiteness of the GS kernel

The fact that the GS kernel is positive semi-definite follows from the following theorem. The proof is provided as supplementary material [see Additional file 1].

##### Theorem 1

Let *Σ* be an alphabet (say the alphabet of all the amino acids). For each *l* ∈ {1, .., *L*}, let $K_{l}:\Sigma^{l}\times \Sigma^{l}\to R$ be a symmetric positive semi-definite kernel. Let A:R→R be any function which consists of a convolution of another function B:R→R by itself. In other words, for all $z,z^{\prime }\in R$, we have

$$A(z-z^{\prime })\;\;=\;\;\int_{-\infty }^{+\infty }\;B(z-t)B(z^{\prime }-t)\;dt\;.$$

Then, the kernel *K* defined, for any two strings of length at least *L* on the alphabet *Σ*, as

$$\begin{array}{lll} K(x,x^{\prime }) & \;\overset{\text{def}}{=}\;\overset{L}{\underset{l=1}{\sum }}\;\overset{|x|-l}{\underset{i=0}{\sum }}\;\overset{|x^{\prime }|-l}{\underset{j=0}{\sum }}A(i-j)\; & \;\\ & \;\;\times K_{l}\left(\left(x_{i+1},\mathrm{..},x_{i+l})\;,\;(x_{j+1}^{\prime },\mathrm{..},x_{j+l}^{\prime }\right)\right)\; & \; \end{array}$$

is also symmetric positive semi-definite.

The positive semi-definiteness of the GS kernel comes from the fact that the GS kernel is a particular case of the more general kernel *K* defined in the above theorem. Indeed, first note that both kernels are identical except *A*(*i* - *j*) in kernel *K* is specialized to $\exp(\frac{-{\left(i-j\right)}^{2}}{2\sigma_{p}^{2}})$ in the GS kernel, and *K*_*l*_(**y**, **y**^′^) in kernel *K* is specialized to $\exp(\frac{-∥\psi^{l}(y)\;-\;\psi^{l}(y){∥}^{2}}{2\sigma_{c}^{2}})$ in the GS kernel. Moreover, this last exponential is just an RBF kernel (see [14] for a definition) defined over vectors of $R^{\text{ld}}$ of the form ***ψ***^*l*^(**y**); it is therefore positive semi-definite for any *l* ∈ {1, 2, .., *L*}. For the first exponential, note that $\exp(\frac{-{\left(i-j\right)}^{2}}{2\sigma_{p}^{2}})$ is a function that is obtained from a convolution of another function since we can verify that

$$\begin{array}{lll} \exp\left(\frac{-{\left(i-j\right)}^{2}}{2\overset{2}{\underset{p}{\sigma }}}\right)\; & =\;\frac{\sqrt{2}}{\sigma_{p}\sqrt{\Pi }}\int_{-\infty }^{+\infty }\exp\left(\frac{-{\left(i-t\right)}^{2}}{\sigma_{p}^{2}}\right)\; & \;\\ & \;\times \exp\left(\frac{-{\left(j-t\right)}^{2}}{\sigma_{p}^{2}}\right)\text{dt}\;.\; & \; \end{array}$$

Indeed, this equality is a simple specialization of Equation (4.13) of [25]. It is related to the fact that the convolution of two Normal distributions is still a Normal distribution.

Finally, it is interesting to point out that Theorem 1 can be generalized to any function *A* on measurable sets *M* (not only the ones that are defined on R), provided that *A* is still is a convolution of another function *B*:*M* → *M*. We omit this generalized version in this paper since Theorem 1 suffices to prove that the GS kernel is positive semi-definite.

#### Efficient computation of the GS kernel

To cope with today’s data deluge, the presented kernel should have a low computational cost. For this task, we first note that, before computing *GS*(**x**, **x**^′^, *L*, *σ*_*p*_, *σ*_*c*_) for each pair (**x**, **x**^′^) in the training set, we can first compute

$$\begin{array}{lll} E(a,a^{\prime })\; & \overset{\text{def}}{=}∥\psi (a)-\psi (a^{\prime }){∥}^{2}\;\; & \;\\ & =\overset{d}{\underset{p=1}{\sum }}{\left(\psi_{p}(a)-\psi_{p}(a^{\prime })\right)}^{2},\; & \; \end{array}$$

for each pair (*a*, *a*^′^) of amino acids. After this pre-computation stage, done in *O*(*d* · |*Σ*|^2^) time, each access to *E*(*a*, *a*^′^) is done in O(1) time. We will not consider the running time of this pre-computation stage in the complexity analysis of the GS kernel, because it only has to be done once to be used for any 5-tuple (**x**, **x**^′^, *L*, *σ*_*p*_, *σ*_*c*_). Recall that the binding affinity predictor, given by Equation 1, can be built only after we have computed the *m*^2^ elements of the kernel matrix **K** (for a training set of *m* examples). Since *m*^2^ is usually much larger than *d* · |*Σ*|^2^, we can omit this pre-computation time in the complexity analysis of kernel evaluations.

Now, recall that we have defined $\psi^{l}:\Sigma^{l}\to R$ as the concatenation of vectors of the form ***ψ***(*a*) (see Equation (2)). Hence, ∥***ψ***^*l*^(**a**) - ***ψ***^*l*^(**a**^′^)∥ is an Euclidian norm, and we have

(5) $$\begin{array}{ll} ∥\psi^{l}(a)-\psi^{l}(a^{\prime }){∥}^{2} & =\overset{l}{\underset{k=1}{\sum }}∥\psi (a_{k})-\psi (a_{k}^{\prime }){∥}^{2}\\ & =\overset{l}{\underset{k=1}{\sum }}E(a_{k},a_{k}^{\prime }) \end{array}$$

Following this, we can now write the GS kernel as

(6) $$\begin{array}{lll} & \text{GS}(x,x^{\prime },L,\sigma_{p},\sigma_{c})\; & \;\\ & \;=\;\overset{L}{\underset{l=1}{\sum }}\overset{|x|-l}{\underset{i=0}{\sum }}\overset{|x^{\prime }|-l}{\underset{j=0}{\sum }}\;e^{\left(\frac{-{\left(i-j\right)}^{2}}{2\sigma_{p}^{2}}\right)}\;e^{\left(\frac{-\overset{l}{\underset{k=1}{\sum }}E(x_{i+k},{x^{\prime }}_{j+k})}{2\sigma_{c}^{2}}\right)}\; \end{array}$$

(7) $$\begin{array}{lll} & \;=\overset{\left|x\right|}{\underset{i=0}{\sum }}\overset{\left|x^{\prime }\right|}{\underset{j=0}{\sum }}\;e^{\left(\frac{-{\left(i-j\right)}^{2}}{2\sigma_{p}^{2}}\right)}\; & \;\\ & \;\times \overset{\min(L,|x|-i,|x^{\prime }|-j)}{\underset{l=1}{\sum }}\;e^{\left(\frac{-\overset{l}{\underset{k=1}{\sum }}E(x_{i+k},{x^{\prime }}_{j+k})}{2\sigma_{c}^{2}}\right)}\;,\; \end{array}$$

where min(*L*, |**x**| - *i*, |**x**^′^| - *j*) is used in order to assure that *i* + *k* and *j* + *k* are valid positions in strings **x** and **x**^′^.

Now, for any *L*, |**x**|, |**x**^′^|, and any *i* ∈ {1, …, |**x**|}, *j* ∈ {1, …, |**x**^′^|}, let

(8) $$B_{i,j}\;\overset{\text{def}}{=}\;\overset{\min(L,|x|-i,|x^{\prime }|-j)}{\underset{l=1}{\sum }}\;e^{\left(\frac{-\overset{l}{\underset{k=1}{\sum }}E(x_{i+k},{x^{\prime }}_{j+k})}{2\sigma_{c}^{2}}\right)}\;.$$

We therefore have

(9) $$\text{GS}(x,x^{\prime },L,\sigma_{p},\sigma_{c})=\overset{\left|x\right|}{\underset{i=0}{\sum }}\overset{\left|x^{\prime }\right|}{\underset{j=0}{\sum }}\exp\left(\frac{-{\left(i-j\right)}^{2}}{2\overset{2}{\underset{p}{\sigma }}}\right)\cdot B_{i,j}\;.$$

Since min(*L*, |**x**| - *i*, |**x**^′^| - *j*) ≤ *L*, we see, from Equation (8), that the computation of each entry *B*_*i*,*j*_ seems to involve *O*(*L*^2^) operations. However, we can reduce this complexity term to *O*(*L*) by a dynamic programming approach. Indeed, consider the following recurrence:

(10) $$t_{k}=\left\{\begin{array}{ll} 1 & \;\;\text{if}\;k=0\\ t_{k-1}\cdot e^{\left(\frac{-E(x_{i+k},{x^{\prime }}_{j+k})}{2\sigma_{c}^{2}}\right)} & \;\;\text{otherwise.} \end{array}\right)$$

We thus have

(11) $$B_{i,j}=\overset{\min(L,|x|-i,|x^{\prime }|-j)}{\underset{k=1}{\sum }}t_{k}$$

The computation of each entry *B*_*i*,*j*_ therefore involves only *O*(*L*) operations. Consequently, the running time complexity of each GS kernel evaluation is *O* (|**x**| · |**x**^′^| · *L*).

To test the efficiency of this dynamic programming algorithm, we conducted an experiment measuring the speedup obtained from using this algorithm versus a naïve implementation of Equation (4) that did not exploit dynamic programming. For peptides of length 15, 35 and 55, we measured the speedup obtained while computing 2,500 kernel values as a function of the kernel parameter *L*.

For a given value of *L*, the speedup *s* is given by *s* = *t*_*n*_ / *t*_*d*_, where *t*_*n*_ is the running time of the naïve implementation and *t*_*d*_ is the running time used by the dynamic programming algorithm.

**Figure 1:**
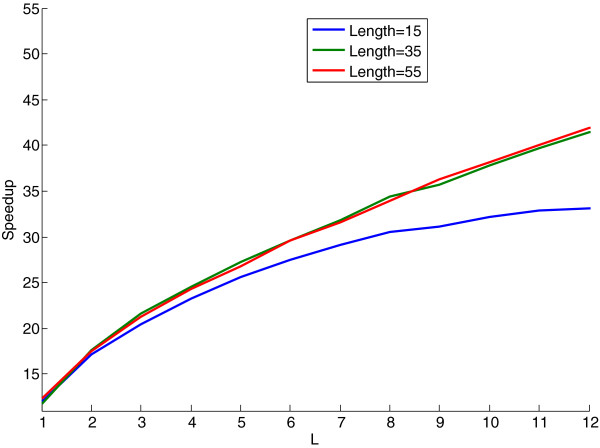
**A benchmark experiment comparing the running times of the GS kernel dynamic programming algorithm and a naïve implementation of the GS kernel.** This figure shows the speedup of the dynamic programming algorithm over a naïve implementation of the GS kernel as a function of the kernel parameter *L*. The running times were recorded while computing 2,500 kernel values for peptides of length 15, 35 and 55. The other kernel parameters are *σ*_*p *_= 0.5 and *σ*_*c *_= 0.5.

The results shown in Figure 1 demonstrate that as the value of *L* increases, the dynamic programming algorithm is much more efficient than the naïve implementation.

#### GS Kernel approximation

In this section, we show how to compute a very close approximation of the GS kernel in linear time. Such a feature is interesting if one wishes to do a pre or post treatment where the symmetric positive semi-definite (SPSD) property of the kernel is not required. For example, as opposed to the training stage where the inverse of **K** + **I** / *C* is guaranteed to exists only for a SPSD matrix **K**, kernel values in the prediction stage could be approximated. Indeed, the scalar product with ***α*** is defined for non positive semi-definite kernel values. This scheme would greatly speed up the predictions with a very small lost of accuracy and precision.

The shifting penalizing term, $\exp\left(\frac{-{\left(i-j\right)}^{2}}{2\sigma_{p}^{2}}\right)$ in Equation (4), implies that the further two substrings are from each other, no matter how similar they are, their contribution to the kernel will vanish exponentially rapidly. Let *δ* be the maximum distance between two substrings that we intend to consider in the computation of the approximate version of the GS kernel. In other words, any substring whose distance is greater than *δ* will not contribute. We propose to fix *δ* = ⌈3*σ*_*p*_⌉. In this case, the contribution of any substring beyond *δ* is bound to be minimal. For the purpose of demonstration, let *P* be the |**x**| × |**x**^′^| matrix

(12) $$P_{i,j}\overset{\text{def}}{=}\left\{\begin{array}{ll} 0 & \;\text{if}\;|i-j|>\delta \\ \exp\left(\frac{-{\left(i-j\right)}^{2}}{2\sigma_{p}^{2}}\right) & \;\text{otherwise}\;. \end{array}\right)$$

*P* is thus a sparse matrix with exactly *δ*|**x**| + *δ*|**x**^′^| - *δ*^2^ non-zero values around it’s diagonal. We can therefore write this approximation of the GS kernel as

(13) $$GS^{\prime }(x,x^{\prime },L,\sigma_{p},\sigma_{c},\delta )=\overset{\left|x\right|}{\underset{i=0}{\sum }}\overset{\left|x^{\prime }\right|}{\underset{j=0}{\sum }}P_{i,j}\cdot B_{i,j}\;.$$

It is clear that only values of *B* for which the value in *P* is non-zero need to be computed. The complexity of *GS*^′^ is dominated by the computation of matrix *B* whose *δ*|**x**| + *δ*|**x**^′^| - *δ*^2^ entries can be computed in *O*(max(|**x**|, |**x**^′^|)). Since *L* and *δ* are constant factors, we have that *GS*^′^ ∈ *O* (max(|**x**|, |**x**^′^|)), giving an optimal linear complexity.

To determine the speedup that can be obtained by approximating the GS kernel, we conducted an experiment measuring this speedup for different peptide lengths. For a given value of *σ*_*p*_, the speedup *s* is given by *s* = *t*_*f*_ / *t*_*a*_, where *t*_*f*_ is the time required for the computation using the GS kernel and *t*_*a*_ is the time required for the computation using the approximated GS kernel.

**Figure 2:**
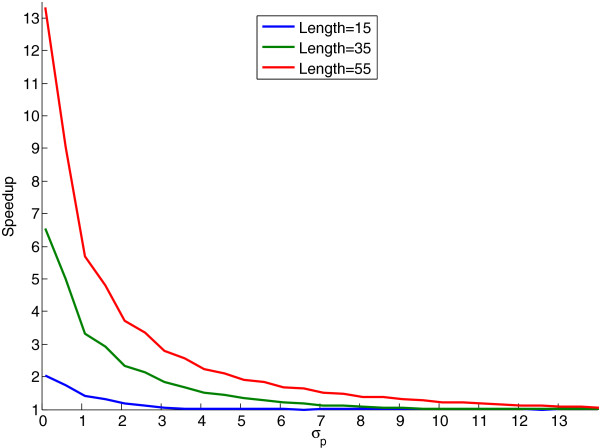
**A benchmark experiment comparing the running times of the approximated GS kernel and the GS kernel.** This figure shows the speedup of the approximation algorithm over the full computation of the GS kernel as a function of the kernel parameter *σ*_*p*_. The running times were recorded while computing 1,000,000 kernel values for peptides of length 15, 35 and 55. The other kernel parameters are *σ*_*c *_= 0.5 and *L *= 5.

Figure 2 displays the speedups obtained for computing 1,000,000 kernel values with peptides of length 15, 35 and 55. We found that the approximation algorithm can greatly reduce the time required to compute kernel values. Note that, since the approximation algorithm only considers substrings of distance less than *δ* = ⌈3*σ*_*p*_⌉, for peptides of length *l*, the speedup obtained by using the approximation algorithm vanishes for *σ*_*p*_ ≥ *l* / 3.

### Kernel for protein binding pocket

**Figure 3:**
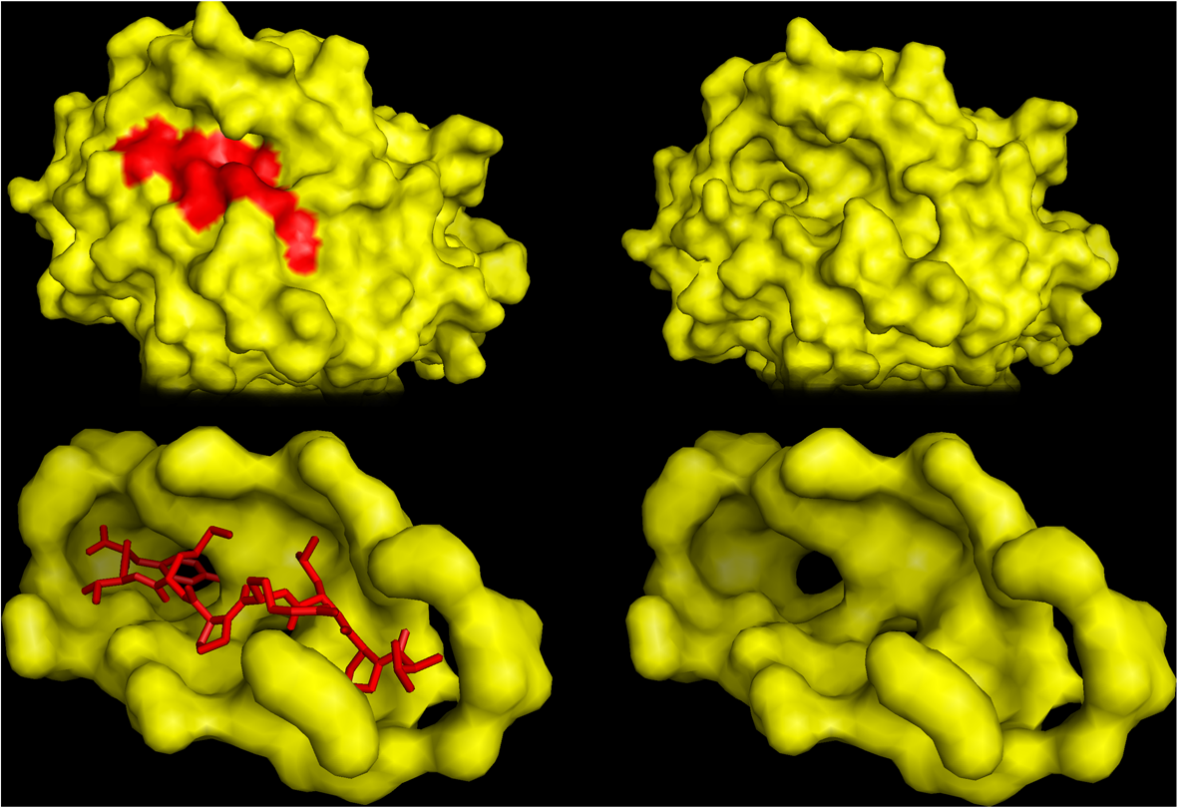
**A pyMOL illustration of a binding pocket used in the binding pocket kernel.** This pyMOL illustration of a binding pocket, used for the binding pocket kernel [26], shows a MHC-I molecule B*3501 complexed with a peptide (VPLRPMTY) from the NEF protein of HIV1 (PDB ID 1A1N). The MHC protein is shown in yellow, the peptide is shown in red.

Hoffmann et al. [26] proposed a new similarity measure between protein binding pockets. The similarity measure aligns atoms extracted from the binding pocket in 3*D* and assigns a score to the alignment. Pocket alignment is possible for proteins that share low sequence and structure similarity. They proposed two variations of the similarity measure. The first variation only compares the shape of pockets to assign a score. In the second variation, atom properties, such as partial charges, re-weight the contribution of each atom to the score. We will refer to these two variations respectively as sup-CK and sup-CK_*L*_. Since both scores are invariant by rotation and translation, they are not positive semi-definite kernels. To obtain a valid kernel, we have used the so-called empirical kernel map where each **y** is mapped explicitly to (*k*(**y**_1_, **y**), *k*(**y**_2_, **y**), …, *k*(**y**_*m*_, **y**)). To ensure reproducibility and avoid implementation errors, all experiments were done using the implementation provided by the authors. An illustration of the pocket creation for the SupCk kernel is shown in Figure 3.

### Kernel for protein structure

The MAMMOTH kernel is a similarity function between protein secondary structure proposed by Qiu et al. [27]. This kernel is based on a sequence-independent structure alignment heuristic originally proposed by Ortiz et al. [28]. Structural information from crystals is used to align two proteins using their secondary structure, a score is assigned to the alignment. The greater the similarity between the two proteins’ secondary structure, the greater the alignment score will be. Ortiz et al. [28] showed that the heuristic was able to produce an accurate alignment for both high and low resolution structures. Also, this kernel was recently used with success for prediction of protein-protein interactions [29]. To ensure reproducibility and avoid implementation errors, all experiments were done using the implementation provided by the authors.

### Metrics and experimental design

When dealing with regression values, classical metrics used for classification such as the area under the ROC curve (AUC) [30] are not suitable. To compute the AUC, some authors determine a binding affinity threshold value and use it to transform the regression problem into a binary classification problem. The real value outputs of the predictor are then mapped to binary classes using the threshold and the AUC is computed using these binary values. Unfortunately, this approach makes the value of the AUC metric dependent on the chosen treshold value. For this reason, we decided not to present results for the AUC metric in this paper. Nevertheless, these results are provided as supplementary material [see Additional file 2].

Fortunately, metrics such as the root mean squared error (RMSE), the coefficient of determination (*R*^2^) and the Pearson product-moment correlation coefficient (PCC) are more suited for measuring the performance of predictors on regression problems. Therefore, in this paper, we have used the PCC and the RMSE to evaluate the performance of our method.

**Figure 4:**
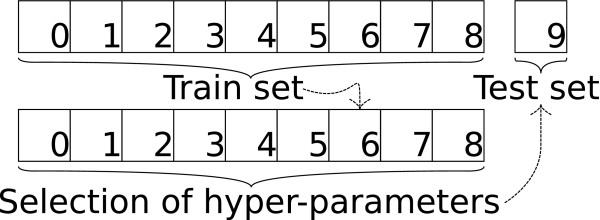
**Illustration of the nested cross-validation procedure.** Nested 10-fold cross-validation. For each of the 10 outer folds, an inner 9 fold cross-validation scheme was used to select hyperparameters.

Except when otherwise stated, 10 folds nested cross-validation was done for estimating the PCC and the RMSE of the predicted binding affinities (See Figure 4). For all *n* (here *n* = 10) outer folds, *n* - 1 inner cross-validation folds were used for the selection of the kernel hyperparameters and the *C* parameter of Equation (1). Note that, all reported values were computed on the union of the outer fold test set predictions. This is important, since an average of correlation coefficients is not a valid correlation coefficient. This is also true for the root mean squared error.

More precisely, let ē denote the average affinity in the data set D. Let *T*_*k*_ for *k* ∈ {1, …, 10} denote the testing set of the *k*^th^ outer fold and let $h_{D\setminus T_{k}}(x_{i},y_{i})$ be the predicted binding affinity on example ((**x**_*i*_, **y**_*i*_), *e*_*i*_) of the predictor built from $D\setminus T_{k}$. The correlation coefficient was computed using:

(14) $$\text{PCC}=\sqrt{1-\frac{\overset{n}{\underset{k=1}{\sum }}\underset{i\in T_{k}}{\sum }{\left(e_{i}-h_{D\setminus T_{k}}(x_{i},y_{i})\right)}^{2}}{\sum_{i\in D}{\left(e_{i}-ē\right)}^{2}}}\;.$$

An algorithm that, on average, produces a predictor that makes the same quadratic error as the constant predictor ē will give *PCC* = 0 and an algorithm that always returns a perfect predictor will give *PCC* = 1.

As for the RMSE, it was computed using

(15) $$\text{RMSE}=\sqrt{\frac{\sum_{k=1}^{n}\sum_{i\in T_{k}}{\left(e_{i}-h_{D\setminus T_{k}}(x_{i},y_{i})\right)}^{2}}{\left|D\right|}}$$

Therefore, the perfect predictor will give *RMSE* = 0 and the value of this metric will increase as the quality of the predictor decrease.

All the p-values reported in this article were computed using the two-tailed Wilcoxon signed-ranked test.

Finally, for all the experiments, hyperparameters for the GS kernels and the learning algorithms were selected by grid search using the following ranges: *C* ∈ ]0, 100], *σ*_*p*_ ∈ ]0, 18], *σ*_*c*_ ∈ [0, 18] and *L* ∈ [1, 15].

### Data

#### PepX database

The PepX database [31] contains 1431 high-quality peptide-protein complexes along with their protein and peptide sequences, high quality crystal structures, and binding energies (expressed in kcal/mol) computed using the FoldX force field method. Full diversity of structural information on protein-peptide complexes is achieved with peptides bound to, among others, MHC, thrombins, *α*-ligand binding domains, SH3 domains and PDZ domains. This database recently drew attention in a review on the computational design of peptide ligands [32] where it was part of large structural studies to understand the specifics of peptide binding. A subset of 505 non-redundant complexes was selected based on the dissimilarity of their binding interfaces. The authors of the database performed the selection in such a way that this smaller subset still represented the full diversity of structural information on peptide-protein complexes present in the entire Protein Data Bank (PDB), see [31] for a description of the method. We will refer to the smaller subset as the “PepX Unique” data set and to the whole data base as “PepX All”.

The few complexes with positive binding energies were removed from the dataset. No other modifications were made to the original database.

#### Major histocompatibility complex class II (MHC-II)

Two different approaches were used for the prediction of MHC class II - peptide binding affinities: single-target and multi-target (pan-specific).

Single-target prediction experiments were conducted using the data from the IEDB dataset proposed by the authors of the RTA method [33]. The latter consists of 16 separate datasets, each containing data on the peptides binding to an MHC class II allotype. For each allotype, the corresponding dataset contains the binding peptide sequences and their binding affinity in kcal/mol. These datasets have previously been separated into 5 cross-validation folds to minimize overlapping between peptide sequences in each fold. It is well known in the machine learning community that such practice should be avoided, as opposed to random fold selection, since the training and test sets should be independently generated. These predefined folds were nevertheless used for the purpose of comparison with other learning methodologies that have used them.

Pan-specific experiments were conducted on the IEDB dataset proposed by the authors of the NetMHCIIpan method [34]. The dataset contains 14 different HLA-DR allotypes, with 483 to 5648 binding peptides per allotype. For each complex, the dataset contains the HLA allele’s identifier (e.g.: DRB1*0101), the peptide’s sequence and the log50*k* transformed IC50 (Inhibitory Concentration 50%), which is given by 1- log50000*IC*50.

As pan-specific learning requires comparing HLA alleles using a kernel, the allele identifiers contained in the dataset were not directly usable for this purpose. Hence, to obtain a useful similarity measure (or kernel) for pairs of HLA alleles, we used the pseudo sequences composed of the amino acids at highly polymorphic positions in the alleles’ sequences. These amino acids are potentially in contact with peptide binders [34], therefore contributing to the MHC molecule’s binding specificity. The authors of the NetMHCIIpan method proposed using pseudo sequences composed of the amino acids at 21 positions that were observed to be polymorphic for HLA-DR, DP and DQ [34]. With respect to the IMGT nomenclature [35], these amino acids are located between positions 1 and 89 of the MHC’s *β* chain. Pseudo sequences consisting of all 89 amino acids between these positions were also used to conduct the experiments.

#### Quantitative structure affinity model (QSAM) benchmark

Three well-studied benchmark datasets for designing quantitative structure affinity models were also used to compare our approach: 58 angiotensin-I converting enzyme (ACE) inhibitory dipeptides, 31 bradykinin-potentiating pentapeptides and 101 cationic antimicrobial pentadecapeptides. These data sets were recently the subject of extensive studies [18] where partial least squares (PLS), Artificial Neural Networks (ANN), Support Vector Regression (SVR), and Gaussian Processes (GP) were used to predict the biological activity of the peptides. GP and SVR were found to have the best results on the testing set, but their experiment protocol was unconventional because the training and test sets were not randomly selected from the data set. Instead, their testing examples were selected from a cluster analysis performed on the whole data set—thus favoring learning algorithms that tend to cluster their predictions according to the same criteria used to split the data. Instead, we randomly selected the testing examples from the whole data set—thus avoiding a bias that would favor some algorithms *a priori*. Theses datasets were chosen to demonstrate the ability of our method to learn on both small and large datasets.

## Results and discussion

### PepX database

To our knowledge, this is the first kernel method attempt at learning a predictor which takes the protein crystal and the peptide sequence as input to predict the binding energy of the complex. Many consider this task as a major challenge with important consequences for molecular biology. Standard string kernels for protein primary structures such as the LA-kernel and the blended spectrum (BS) were used while conducting experiments on proteins. They did not yield good results, mainly because they do not consider the protein’s secondary structure information. To validate this hypothesis and improve our results, we tried using the MAMMOTH kernel. The MAMMOTH kernel did improve the results (see Table 2) over the blended spectrum (BS) but was still missing an important aspect of protein-peptide interaction. The interaction takes place at a very specific location on the surface of the protein called the binding pocket. Two proteins may be very different, but if they share a common binding pocket, it is likely that they will bind similar ligands. This is the core idea that motivated the design of the sup-CK binding pocket kernel [26].

**Table 2:** Correlation coefficient (PCC) for multiple target predictions on the PepX database

	**SVR**	**KRR**					
	**sup-CK**	**sup-CK**		**BS**	**MAMMOTH**	**sup-CK**_***L***_	
	**BS**	**BS**	**GS**	**BS**	**BS**	**BS**	**GS**
PepX Unique	0.6822	0.7072	**0.7300**	0.5873	0.5828	0.7110	0.7264
PepX All	0.8227	0.8580	0.8648	0.7769	0.8152	0.8601	**0.8652**

Best results are highlighted in bold.

Choosing a kernel for the peptides was also a challenging task. Sophisticated kernels for local signals such as the RBF, the weighted degree, and the weighted degree RBF could not be used because peptide lengths were not equal. In fact, peptide lengths vary between 5 and 35 amino acids, which makes the task of learning a predictor and designing a kernel even more challenging. This was part of our motivation in designing the GS kernel. For all experiments, the BLOSUM 50 matrix was found to be the optimal amino acid descriptors during cross-validation.

Table 2 presents the first machine learning results for the prediction of binding affinity given any peptide-protein pair. We first observe that KRR has better accuracy than SVR. We also note that using the GS kernel over the simpler BS kernel improves the accuracy for both the sup-CK and the sup-CK_*L*_ kernels for binding pockets. It is surprising that the sup-CK_*L*_ kernel does not outperform the sup-CK kernel on both benchmarks, since the addition of the atom partial charges should provide more relevant information to the predictor.

**Figure 5:**
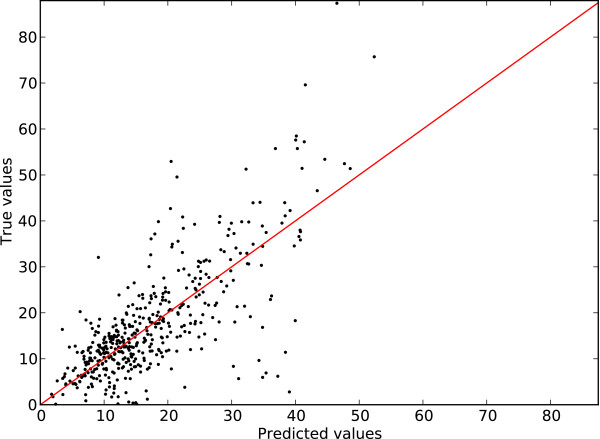
**Predicted values as a function of the true values for the PepX Unique dataset.** Predicted values for all peptide-protein complexes as a function of the true value. A perfect predictor would have all it’s predictions lying on the *y* = *x* red line.

**Figure 6:**
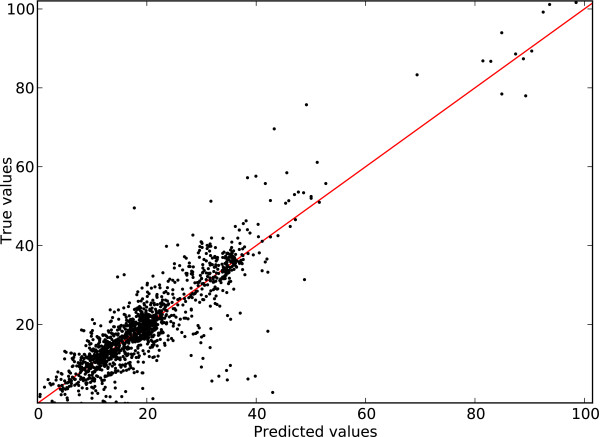
**Predicted values as a function of the true values for the PepX All dataset.** Predicted values for all peptide-protein complexes as a function of the true value. A perfect predictor would have all it’s predictions lying on the *y* = *x* red line.

Figures 5 and 6 present an illustration of the prediction accuracy using sup-CK for the PepX Unique dataset and sup-CK_*L*_ for the PepX All dataset. For illustration purposes, the absolute value of the binding energy has been plotted. We observe that the predictor has the property of maintaining ranking of binding affinities. Consequently, peptides with high binding affinity can generally be identified—an important feature for drug discovery. Peptides with the highest binding affinities are the ones that, ultimately, will serve as precursor drug or scaffold in a rational drug design program.

Experiments showed that a Pearson correlation coefficient of ≈1.0 is attainable on the training set when using the binding pocket kernel, the GS kernel and a large value for the complexity-accuracy trade-off parameter *C* (empirically ≈100), thus giving little weight to the regularization term. This is a strong indication that the proposed method has the ability of building a good predictor, but the lack of data quality and quantity may be responsible for the reduced performance on the testing set. Hence better data may improve the quality of the predictor. Initially, biological validation will be necessary but ultimately, when sufficient data is gathered, the predictor may provide accurate results that are currently only achievable by high cost biological experimentation.

### Major histocompatibility complex class II (MHC-II)

#### Single-target predictions

We performed a single-target prediction experiment using the dataset proposed by the authors of the RTA method [33]. The goal of such experiments was to evaluate the ability of a predictor to predict the binding energy (kcal/mol) of an unknown peptide to a specific MHC allotype when training only on peptides binding to this allotype. For each of the 16 MHC allotypes, a predictor was trained using kernel ridge regression with the GS kernel and a nested cross-validation scheme was used. For comparison purposes, the nested cross-validation was done using the 5 predefined cross-validation folds provided in [33]. Again, this is sub-optimal from the statistical machine learning perspective, since the known guarantees on the risk of a predictor [14,19] normally require that the examples be generated independently from the same distribution.

Three common metrics were used to compare the methods: the Pearson correlation coefficient (PCC), the root mean squared error (RMSE), and the area under the ROC curve (AUC). The PCC and the RMSE results are presented in Table 3, AUC values can be found as supplementary material [see Additional file 2]. The PCC results show that our method significantly outperforms the RTA method on 13 out of 16 allotypes with a p-value of 0.0308. The inferior results for certain allotypes may be attributed to the small size of these datasets. In addition, the RMSE results show that our method clearly outperforms the RTA method on all 16 allotypes with a p-value of 0.0005.

**Table 3:** Comparison of HLA-DR prediction results on the dataset proposed by the authors of RTA

	**PCC**		**RMSE (kcal/mol)**		
**MHC *β *chain**	**KRR+GS**	**RTA**	**KRR+GS**	**RTA**	**# of examples**
DRB1*0101	**0.632**	0.530	**1.20**	1.43	5648
DRB1*0301	**0.538**	0.425	**1.16**	1.46	837
DRB1*0401	**0.430**	0.340	**1.44**	1.72	1014
DRB1*0404	**0.491**	0.487	**1.25**	1.38	617
DRB1*0405	**0.530**	0.442	**1.09**	1.35	642
DRB1*0701	**0.645**	0.484	**1.24**	1.62	833
DRB1*0802	**0.469**	0.412	**1.19**	1.34	557
DRB1*0901	0.303	**0.369**	**1.55**	1.68	551
DRB1*1101	**0.550**	0.450	**1.17**	1.45	812
DRB1*1302	**0.468**	0.464	**1.51**	1.64	636
DRB1*1501	**0.502**	0.438	**1.41**	1.53	879
DRB3*0101	0.380	**0.425**	**1.03**	1.13	483
DRB4*0101	**0.613**	0.522	**1.10**	1.33	664
DRB5*0101	**0.541**	0.434	**1.20**	1.57	835
H2*IA_*b*_	**0.603**	0.556	**1.00**	1.15	526
H2*IA_d_	0.325	**0.563**	**1.44**	1.53	306
Average:	**0.501**	0.459	**1.25**	1.46	

Best results for each metric are highlighted in bold. The PCC results show that the proposed method (KRR+GS) outperforms the RTA method with a p-value of 0.0308. The RMSE results show that KRR+GS outperforms the RTA method on all 16 allotypes with a p-value of 0.0005.

#### Pan-specific predictions

To evaluate the performance of our method and the potential of the GS kernel, pan-specific predictions were performed using the dataset proposed by the authors of NetMHCIIpan [34]. The authors proposed a new cross-validation scheme called the *leave one allele out* (LOAO) where all but one allele are used as training set and the remaining allele is used as testing set. This is a more challenging problem, as the predictor needs to determine the binding affinity of peptides for an allele which was absent in the training data. The binding specificity of an allele’s interface is commonly characterized using a pseudo sequence extracted from the beta chain’s sequence [11,13,34]. During our experiments, the 21 amino acid pseudo sequences were found to be optimal. The 89 amino acid pseudo sequences yielded similar, but slightly suboptimal results. For all experiments, the GS kernel was used for the allele pseudo sequences and for the peptide sequences. All results were obtained with the same LOAO scheme presented in [34]. For each allele, an inner LOAO cross-validation was done for the selection of hyperparameters.

To assess the performance of the proposed method, the PCC and the RMSE results are shown in Table 4, AUC values can be found in the supplementary material [see Additional file 2]. Since we performed LOAO cross-validation, the PCC, RMSE and AUC values were calculated on each test fold individually, thus yielding results for each allele.

**Table 4:** Comparison of pan-specific HLA-DR prediction results on the dataset proposed by the authors of NetMHCIIpan

	**PCC**			**RMSE (kcal/mol)**		
**MHC *β *chain**	**KRR+GS**	**MultiRTA**	**NetMHCIIpan-2.0**	**KRR+GS**	**MultiRTA**	**# of examples**
DRB1*0101	**0.662**	0.619	0.627	1.48	**1.33**	5166
DRB1*0301	**0.743**	0.438	0.560	**1.29**	1.36	1020
DRB1*0401	**0.667**	0.534	0.652	**1.36**	1.56	1024
DRB1*0404	0.709	0.623	**0.731**	**1.18**	1.33	663
DRB1*0405	0.606	0.566	**0.626**	**1.25**	1.28	630
DRB1*0701	0.694	0.620	**0.753**	**1.34**	1.51	853
DRB1*0802	**0.728**	0.523	0.700	**1.23**	1.45	420
DRB1*0901	0.471	0.375	**0.474**	**1.53**	2.01	530
DRB1*1101	**0.786**	0.603	0.721	**1.16**	1.46	950
DRB1*1302	**0.416**	0.365	0.337	1.73	**1.68**	498
DRB1*1501	**0.612**	0.513	0.598	**1.46**	1.57	934
DRB3*0101	**0.654**	0.603	0.474	1.52	**1.10**	549
DRB4*0101	**0.540**	0.508	0.515	**1.41**	1.61	446
DRB5*0101	**0.732**	0.543	0.722	**1.28**	1.60	924
Average:	**0.644**	0.531	0.606	**1.37**	1.49	

Best results for each metric are highlighted in bold. The PCC results show that the proposed method (KRR+GS) outperforms MultiRTA with a p-value of 0.001 and NetMHCIIpan-2.0 with a p-value of 0.0574. The RMSE results indicate that KRR+GS outperforms MultiRTA with a p-value of 0.0466.

The PCC results show that our method outperforms the MultiRTA [12] (p-value of 0.001) and the NetMHCIIpan-2.0 [13] (p-value of 0.0574) methods. Since the dataset contained values in log50*k* transformed IC50 (Inhibitory Concentration 50%), the calculation of the RMSE values required converting the predicted values to kcal/mol using the method proposed in [33].

The RMSE values are only shown for our method and the MultiRTA method, since such values were not provided by the authors of NetMHCIIpan-2.0. The RMSE results indicate that our method globally outperforms MultiRTA with a p-value of 0.0466.

### Quantitative structure affinity model (QSAM) benchmark

For all datasets, the extended *z* scale [18] was found to be the optimal amino acids descriptors during cross-validation. All the results in this section were thus obtained using the extended *z* scale for the RBF and GS kernels. All peptides within each data set are of the same length, which is why the RBF kernel can be applied, as opposed to the PepX database or the two MHC-II benchmark datasets. Note the RBF kernel is a special case of the GS kernel. Hence, the results obtained from our method using the GS kernel were likely to be at least as good as those obtained with the RBF kernel.

Table 5 present the results obtained when applying the method from [18] (SVR learning with the RBF kernel) and our method (KRR learning with the GS kernel). Results with the RBF kernel and KRR are also presented to illustrate the gain in accuracy obtained from the more general GS kernel.

**Table 5:** Correlation coefficient (PCC) on the QSAM benchmarks

	**SVR**	**KRR**	
	**RBF**	**RBF**	**GS**
ACE	0.8782	0.8807	**0.9044**
Bradykinin	0.7491	0.7531	**0.7641**
Cationic	0.7511	0.7417	**0.7547**

Best results are highlighted in bold.

We observed that kernel ridge regression (KRR) had a slight accuracy advantage over support vector regression (SVR). Moreover, SVR has one more hyperparameter to tune than KRR: the *ϵ*-insensitive parameter. Consequently, KRR should be preferred over SVR for requiring a substantially shorter learning time. Also, we show in Table 5 that the GS kernel outperforms the RBF kernel on all three QSAM data sets (when limiting ourself to KRR). Considering these results, KRR with the GS kernel clearly outperforms the method of [18] on all data sets.

### Additionnal results and external validation

To act as an external source of validation for our results and to assess the performance of the GS kernel, we participated in the 2012 Machine Learning Competition in Immunology [36]. The goal of this competition was to identify, given unpublished experimental data, which new peptides were naturally processed by MHC Class I pathway for 8 target molecules. Our method achieved the best prediction performance for HLA-B*0702, HLA-B*5301, H2-Db, and H2-Kb molecules, validating the suitability of the GS kernel for such problems.

These results support our claim that the GS kernel is a state-of-the-art kernel for peptides and a valuable tool for computationnal biologists.

## Conclusions

We have proposed a new kernel designed for small bio-molecules (such as peptides) and pseudo-sequences of binding interfaces. The GS kernel is an elegant generalization of eight known kernels for local signals. Despite the richness of this new kernel, we have provided a simple and efficient dynamic programming algorithm for its exact computation and a linear time algorithm for its approximation. Combined with the kernel ridge regression learning algorithm and the binding pocket kernel, the proposed kernel yields promising results on the PepX database. For the first time, a predictor capable of accurately predicting the binding affinity of any peptide to any protein was learned using this database. Our method significantly outperformed RTA on the single-target prediction of MHC-II binding peptides. Impressive state-of-the-art results were also obtained on the pan-specific MHC-II task, outperforming both MultiRTA and NetMHCIIpan-2.0. Moreover, the method was successfully tested on three well studied datasets for the quantitative structure affinity model.

A predictor trained on the whole IEDB database or PDB database, as opposed to benchmark datasets, would be a substantial tool for the community. Unfortunately, learning a predictor on very large datasets (over 2,5000 examples) is still a major challenge with most machine learning methods, as the similarity (Gram) matrix becomes hard to fit into the memory of most computers. We propose to expand the presented method to very large datasets as future work. The proposed kernel is freely available at http://graal.ift.ulaval.ca/downloads/gs-kernel/.

## Competing interests

The authors declare that they have no competing interests.

## Authors’ contributions

SG designed the GS kernel, algorithms for it’s computation, implemented the learning algorithm and conducted experiments on the PepX and QSAM datasets. MM designed the learning algorithm. FL and MM did the proof of the symmetric positive semi-definiteness of the GS kernel. AD conducted experiments on MHC-II datasets. JC provided biological insight and knowledge. This work was done under the supervision of MM, FL and JC. All authors contributed to, read and approved the final manuscript.

## Supplementary Material

Additional file 1 **The proof of theorem 1.** This file presents the proof of Theorem 1, therefore it proves that the GS kernel is symmetric positive semi-definite. Click here for file

Additional file 2 **AUC results for experiments on MHC-II.** This file presents AUC values obtained for the experiments on MHC-II datasets and provides an explanation on how these values were calculated. Click here for file
