# Supplementary material for: Learning a peptide-protein binding affinity predictor with kernel ridge regression
Source: BMC Bioinformatics. 2013 Mar 5;14:82. doi: 10.1186/1471-2105-14-82 (PMC3651388; doi:10.1186/1471-2105-14-82)
Supplement: Additional file 1 — The proof of theorem 1. This file presents the proof of Theorem 1, therefore it proves that the GS kernel is symmetric positive semi-definite. [file 1471-2105-14-82-S1.pdf]

1 The proof of theorem 1

Theorem 1 *Let Σ be an alphabet (say the alphabet of all the amino acids). For each $l \in \{1,..,L\}$, let $K_l : \Sigma^l \times \Sigma^l \longrightarrow \mathbb{R}$ be a symmetric positive semi-definite kernel. Let $A : \mathbb{R} \longrightarrow \mathbb{R}$ be any function which consists of a convolution of another function $B : \mathbb{R} \longrightarrow \mathbb{R}$ by itself. In other words, for all $z, z' \in \mathbb{R}$, we have*

$$A(z - z') \quad = \quad \int_{-\infty}^{+\infty} B(z-t)B(z'-t)\,\mathrm{d}t\,.$$

Then, the kernel K defined, for any two strings of length at least L on the alphabet Σ, as

$$K(\mathbf{x},\mathbf{x}') \quad \stackrel{def}{=} \quad \sum_{l=1}^{L}\sum_{i=0}^{|\mathbf{x}|-l}\sum_{j=0}^{|\mathbf{x}'|-l} A(i-j)\ K_l\Big((x_{i+1},..,x_{i+l})\,,\,(x'_{j+1},..,x'_{j+l})\Big)$$

is also symmetric positive semi-definite.

Let us first recall the well-known definition (due to Mercer (1909)) of a PSD kernel that we state here in its "string kernel" version. See also [1] for a similar formulation.

Definition 2 [Mercer] Let Σ be a finite alphabet and $\mathcal{D} \subseteq \Sigma^\star$, a set of words on that alphabet. Then, a symmetric string kernel $\tilde{K} : \mathcal{D}\times\mathcal{D} \longrightarrow \mathbb{R}$ is *positive semi-definite* if and only if it satisfies Mercer's condition, *i.e.*,

$$\sum_{\mathbf{x}\in\mathcal{D}}\sum_{\mathbf{x}'\in\mathcal{D}} f(\mathbf{x})f(\mathbf{x}')\tilde{K}(\mathbf{x},\mathbf{x}') \quad \geq \quad 0$$

for any function $f : \mathcal{D} \longrightarrow \mathbb{R}$ such that $\sum_{\mathbf{x}\in\mathcal{D}} f^2(\mathbf{x}) < +\infty\,.$

Proof of Theorem 1: For compactness of the notation, the sub-string $(x_{i+1}, x_{i+2}, \ldots, x_{i+l})$ of a string $\mathbf{x}$ is denoted $\mathbf{x}_{]i:i+l]}$. Also, the set of all strings of of length at least (respectively at most) k on the alphabet Σ is denoted $\Sigma^{\geq k}$ (respectively $\Sigma^{\leq k}$). Clearly, the kernel K is symmetric and has domain $\Sigma^{\geq L} \times \Sigma^{\geq L}$. So, we only have to show that it satisfies Mercer's condition of Definition 2 (with $\mathcal{D} = \Sigma^{\geq L}$ and $\tilde{K} = K$). Consequently, we have

$$\begin{aligned}
\sum_{\mathbf{x}\in\Sigma^{\geq L}}\sum_{\mathbf{x}'\in\Sigma^{\geq L}} f(\mathbf{x})f(\mathbf{x}')K(\mathbf{x},\mathbf{x}') \;&=\; \sum_{s=L}^{+\infty}\sum_{s'=L}^{+\infty}\sum_{\mathbf{x}\in\Sigma^{s}}\sum_{\mathbf{x}'\in\Sigma^{s'}} f(\mathbf{x})f(\mathbf{x}')K(\mathbf{x},\mathbf{x}') \\
&=\; \sum_{s=L}^{+\infty}\sum_{s'=L}^{+\infty}\sum_{\mathbf{x}\in\Sigma^{s}}\sum_{\mathbf{x}'\in\Sigma^{s'}}\sum_{l=1}^{L}\sum_{i=0}^{|\mathbf{x}|-l}\sum_{j=0}^{|\mathbf{x}'|-l} A(i-j)\ f(\mathbf{x})f(\mathbf{x}')K_l(\mathbf{x}_{]i,i+l]},\mathbf{x}'_{]j,j+l]}) \\
&=\; \sum_{s=L}^{+\infty}\sum_{s'=L}^{+\infty}\sum_{\mathbf{x}\in\Sigma^{s}}\sum_{\mathbf{x}'\in\Sigma^{s'}}\sum_{l=1}^{L}\sum_{i=0}^{s-l}\sum_{j=0}^{s'-l} A(i-j)\ f(\mathbf{x})f(\mathbf{x}')K_l(\mathbf{x}_{]i,i+l]},\mathbf{x}'_{]j,j+l]}) \\
&=\; \sum_{l=1}^{L}\sum_{s=L}^{+\infty}\sum_{s'=L}^{+\infty}\sum_{i=0}^{s-l}\sum_{j=0}^{s'-l} A(i-j)\ \sum_{\mathbf{x}\in\Sigma^{s}}\sum_{\mathbf{x}'\in\Sigma^{s'}} f(\mathbf{x})f(\mathbf{x}')K_l(\mathbf{x}_{]i,i+l]},\mathbf{x}'_{]j,j+l]})
\end{aligned} \tag{$\star$}$$

Note that the last part of the last line of Equation ($\star$) can be rewritten as

$$
\begin{aligned}
& A(i-j) \sum_{\mathbf{x}\in\Sigma^s} \sum_{\mathbf{x}'\in\Sigma^{s'}} f(\mathbf{x}) f(\mathbf{x}') K_l(\mathbf{x}_{]i,i+l]}, \mathbf{x}'_{]j,j+l]}) \\
&\quad = A(i-j) \sum_{\mathbf{y}\in\Sigma^l} \sum_{\mathbf{y}'\in\Sigma^l} \sum_{\mathbf{x}\in\Sigma^s | \mathbf{x}_{]i:i+l]}=\mathbf{y}} \sum_{\mathbf{x}'\in\Sigma^{s'} | \mathbf{x}'_{]i:i+l]}=\mathbf{y}'} f(\mathbf{x}) f(\mathbf{x}') K_l(\mathbf{y}, \mathbf{y}') \\
&\quad = \int_{-\infty}^{+\infty} B(i-t) B(j-t)\, \mathrm{d}t \sum_{\mathbf{y}\in\Sigma^l} \sum_{\mathbf{y}'\in\Sigma^l} \sum_{\mathbf{x}\in\Sigma^s | \mathbf{x}_{]i:i+l]}=\mathbf{y}} \sum_{\mathbf{x}'\in\Sigma^{s'} | \mathbf{x}'_{]i:i+l]}=\mathbf{y}'} f(\mathbf{x}) f(\mathbf{x}') K_l(\mathbf{y}, \mathbf{y}')\,, \qquad (\star\star)
\end{aligned}
$$

where $\mathbf{x} \in \Sigma^s \mid \mathbf{x}_{]i:i+l]} = \mathbf{y}$ means that the strings $\mathbf{x}$ is any string of length s that contains the substring $\mathbf{y}$ starting at position $i+1$.

Now, for any integers $s \geq L$ and $l \geq 1$, let us define $g_{s,l} : \Sigma^l \times \mathbb{R} \longrightarrow \mathbb{R}$, as

$$
g_{s,l}(\mathbf{y}, t) \stackrel{\text{def}}{=} \sum_{i=0}^{s-l} B(i-t) \sum_{\mathbf{x}\in\Sigma^s | \mathbf{x}_{]i:i+l]}=\mathbf{y}} f(\mathbf{x})\,. \qquad (1)
$$

Note that $g_{s,l}(\mathbf{y}, t)$ is finite because it is a double sum, each of which being finite. It therefore follows follows that

$$
\sum_{\mathbf{y}\in\Sigma^l} [g_{s,l}(\mathbf{y}, t)]^2 \ < \ \infty\,, \qquad (\star\star\star)
$$

because this is also a finite sum. Now, combining ($\star$) and ($\star\star$), we obtain

$$
\begin{aligned}
& \sum_{\mathbf{x}\in\Sigma^{\geq L}} \sum_{\mathbf{x}'\in\Sigma^{\geq L}} f(\mathbf{x}) f(\mathbf{x}') K(\mathbf{x}, \mathbf{x}') \\
&\quad = \sum_{l=1}^{L} \sum_{(\mathbf{y},\mathbf{y}')\in\Sigma^l\times\Sigma^l} K_l(\mathbf{y}, \mathbf{y}') \sum_{s=L}^{+\infty} \sum_{i=0}^{s-l} \sum_{s'=L}^{+\infty} \sum_{j=0}^{s'-l} \int_{-\infty}^{+\infty} B(i-t) B(j-t)\, \mathrm{d}t \\
&\qquad\qquad \times \left[\sum_{\mathbf{x}\in\Sigma^s | \mathbf{x}_{]i:i+l]}=\mathbf{y}} f(\mathbf{x}) \right] \left[\sum_{\mathbf{x}'\in\Sigma^{s'} | \mathbf{x}'_{]j:j+l]}=\mathbf{y}'} f(\mathbf{x}') \right] \\
&\quad = \sum_{l=1}^{L} \sum_{s=L}^{+\infty} \sum_{s'=L}^{+\infty} \int_{-\infty}^{+\infty} \left(\sum_{(\mathbf{y},\mathbf{y}')\in\Sigma^l\times\Sigma^l} K_l(\mathbf{y}, \mathbf{y}') \left[\sum_{i=0}^{s-l} B(i-t) \sum_{\mathbf{x}\in\Sigma^s | \mathbf{x}_{]i:i+l]}=\mathbf{y}} f(\mathbf{x}) \right] \right. \\
&\qquad\qquad \left. \times \left[\sum_{j=0}^{s'-l} B(j-t) \sum_{\mathbf{x}'\in\Sigma^{s'} | \mathbf{x}'_{]j:j+l]}=\mathbf{y}'} f(\mathbf{x}') \right] \right) \mathrm{d}t \\
&\quad = \sum_{l=1}^{L} \sum_{s=L}^{+\infty} \sum_{s'=L}^{+\infty} \int_{-\infty}^{+\infty} \left(\sum_{(\mathbf{y},\mathbf{y}')\in\Sigma^l\times\Sigma^l} K_l(\mathbf{y}, \mathbf{y}') g_{s,l}(\mathbf{y}, t) g_{s',l}(\mathbf{y}', t) \right) \mathrm{d}t \\
&\quad \geq 0\,.
\end{aligned}
$$

The last line follows from Definition 2 (with $\mathcal{D} = \Sigma^l$, and $\tilde{K} = K_l$), Equation ($\star\star\star$) and the fact that, by hypothesis, K_l is PSD. Consequently, K is PSD. $\square$

References

1. Cristianini N, Shawe-Taylor J: *An Introduction to Support Vector Machines and Other Kernel-Based Learning Methods*. Cambridge, U.K.: Cambridge University Press 2000.
