# Supplementary material for: Learning a peptide-protein binding affinity predictor with kernel ridge regression
Source: BMC Bioinformatics. 2013 Mar 5;14:82. doi: 10.1186/1471-2105-14-82 (PMC3651388; doi:10.1186/1471-2105-14-82)
Supplement: Additional file 2 — AUC results for experiments on MHC-II. This file presents AUC values obtained for the experiments on MHC-II datasets and provides an explanation on how these values were calculated. [file 1471-2105-14-82-S2.pdf]

1 AUC results for experiments on MHC-II

Calculating the area under the ROC curve (AUC) [1] for regression problems requires transforming the initial problem into a classification problem [2]. In the case of MHC-peptide binding affinities, the classification problem aims to distinguish between binders and non-binders. To achieve this, a threshold value allowing to distinguish binders from non-binders is required.

1.1 Single-target experiment

For this experiment, the predicted values were binding energies in kcal/mol. As proposed in [2], we set a binding affinity threshold of 500nM. Therefore, the threshold value (t) in nanomolar was converted to kcal/mol using the technique proposed in [3]:

$$t = -0.586 \times log(500 \times 10^{-9}) = 8.50207 kcal/mol.$$

To calculate the AUC, we converted all the binding energies in the dataset to binary classes based on this binding threshold. Then, for all examples, we predicted the binding energy value (e) and generated a confidence value that the given example was a binder. This confidence value is given by: $c = e - t$ and then normalized using all other confidence values to be in the range $[0, 1]$. The latter were used to calculate the AUC for the experiment.

Table 1: Results of the comparison between AUC values for binding energy predictions obtained from Kernel Ridge Regression and the GS Kernel versus the RTA [3] method on the dataset proposed by the authors of this method.

	**AUC**		
**MHC β chain**	**KRR+GS**	**RTA**	**# of examples**
DRB1*0101	**0.838**	0.749	5648
DRB1*0301	**0.781**	0.762	837
DRB1*0401	**0.753**	0.715	1014
DRB1*0404	0.786	**0.792**	617
DRB1*0405	**0.782**	0.757	642
DRB1*0701	**0.845**	0.790	833
DRB1*0802	0.724	**0.747**	557
DRB1*0901	0.665	**0.711**	551
DRB1*1101	**0.830**	0.753	812
DRB1*1302	0.708	**0.765**	636
DRB1*1501	**0.740**	0.736	879
DRB3*0101	0.716	**0.825**	483
DRB4*0101	**0.831**	0.799	664
DRB5*0101	**0.826**	0.732	835
H2*IA$_b$	**0.860**	0.828	526
H2*IA$_d$	0.772	**0.814**	306
Average:	**0.779**	0.767	

1.2 Pan-specific experiment

The AUC for this experiment was calculated using confidence values as explained above. A threshold of 500 nM was used to discriminate binders from non-binders [2].

Table 2: Results of the comparison between AUC values for binding affinity predictions obtained from Kernel Ridge Regression and the GS Kernel versus the MultiRTA [4] and the NetMHCIIpan-2.0 [5] methods on the dataset proposed by the authors of NetMHCIIpan [2].

	AUC			
**MHC β chain**	**KRR+GS**	**MultiRTA**	**NetMHCIIpan-2.0**	**# of examples**
DRB1*0101	**0.807**	0.801	0.794	5166
DRB1*0301	0.775	0.751	**0.792**	1020
DRB1*0401	**0.802**	0.763	**0.802**	1024
DRB1*0404	0.862	0.835	**0.869**	663
DRB1*0405	**0.827**	0.808	0.823	630
DRB1*0701	**0.891**	0.817	0.886	853
DRB1*0802	0.840	0.786	**0.869**	420
DRB1*0901	**0.685**	0.674	0.684	530
DRB1*1101	**0.900**	0.819	0.875	950
DRB1*1302	0.648	**0.698**	0.648	498
DRB1*1501	**0.773**	0.729	0.769	934
DRB3*0101	0.690	**0.813**	0.733	549
DRB4*0101	0.759	0.746	**0.762**	446
DRB5*0101	**0.888**	0.788	0.879	924
Average:	0.796	0.773	**0.800**	

References

1. Swets J: **Measuring the accuracy of diagnostic systems**. *Science* 1988, **240**(4857):1285–1293, [http://www.sciencemag.org/content/240/4857/1285.abstract].

2. Nielsen M, Lundegaard C, Blicher T, Peters B, Sette A, Justesen S, Buus S, Lund O: **Quantitative Predictions of Peptide Binding to Any HLA-DR Molecule of Known Sequence: NetMHCIIpan**. *PLoS Comput Biol* 2008, **4**(7):e1000107, [http://dx.plos.org/10.1371%2Fjournal.pcbi.1000107].

3. Bordner AJ, Mittelmann HD: **Prediction of the binding affinities of peptides to class II MHC using a regularized thermodynamic model**. *BMC Bioinformatics* 2010, **11**:41, [www.biomedcentral.com/1471-2105/11/41].

4. Bordner AJ, Mittelmann HD: **MultiRTA: A simple yet reliable method for predicting peptide binding affinities for multiple class II MHC allotypes.** *BMC Bioinformatics* 2010, **11**:482, [dblp.uni-trier.de/db/journals/bmcbi/bmcbi11.html#BordnerM10a].

5. Nielsen M, Justesen S, Lund O, Lundegaard C, Buus S: **NetMHCIIpan-2.0 - Improved pan-specific HLA-DR predictions using a novel concurrent alignment and weight optimization training procedure**. *Immunome Research* 2010, **6**:9, [http://www.immunome-research.com/content/6/1/9].
